# Treatment effects of a fixed intermaxillary device to correct class II malocclusions in growing patients

**DOI:** 10.1186/s40510-014-0045-x

**Published:** 2014-08-01

**Authors:** Dean A Heinrichs, Imad Shammaa, Chris Martin, Thomas Razmus, Erdogan Gunel, Peter Ngan

**Affiliations:** Dr. Heinrichs postal code, Saskatoon, SK S7H3B9 Canada; Charleston, WV 26506 USA; Department of Orthodontics, School of Dentistry, West Virginia University, 1073 Health Science Center North, P.O. Box 9480, Morgantown, WV 26506 USA; Department of Periodontics and Oral Medicine, School of Dentistry, University of Michigan, 1011 North University Avenue, Ann Arbor, MI 48109 USA; Department of Statistics, School of Dentistry, West Virginia University, Morgantown, WV 26506 USA

## Abstract

**Background:**

The objective of this study was to evaluate the treatment effects of Forsus™ Fatigue Resistant Device (FRD; 3M Unitek, Monrovia, CA, USA) in growing patients with Class II non-extraction malocclusions.

**Methods:**

A retrospective sample of 24 class II patients treated consecutively with the FRD followed by comprehensive orthodontic treatment was compared to a sample of untreated control subjects from the Bolton Brush Study who was matched in age, sex, and craniofacial morphology. Lateral cephalometric radiographs were taken before treatment (T1) and after removal of fixed appliances (T2). Growth changes were subtracted from the treatment changes to obtain the treatment effects of the appliance. Data were analyzed using ANOVA and a match paired *t* test.

**Results:**

Significant differences were found between the treated and control groups for 12 of the 29 measured variables (Co-Gn minus Co-Apt, Wits, Is-OLp, Ii-OLp, overjet, Mi-OLp, molar relationship, overbite, Mic-ML, SNA, ANB, and Ii-ML). With 27.8 months of treatment, all patients were corrected to a class I dental arch relationship. Overjet and molar relationships were improved by an average of 4.7 and 3.1 mm, respectively. This was contributed by a 1.2 mm of restraint in forward maxillary growth, 0.7 mm of forward movement of the mandible, 1.5 mm of backward movement of the maxillary incisors, 1.3 mm forward movement of the mandibular incisors, 0.5 mm backward movement of the maxillary molars, and 1.3 mm of forward movement of the mandibular molars. The overbite was decreased by 2 mm with no significant change in the occlusal, palatal, or mandibular plane. Individual variations in response to the FRD treatment were large for most of the parameters tested. Significant differences in treatment changes between male and female subjects were found only in a few parameters measured.

**Conclusions:**

These results demonstrate that significant overjet and overbite corrections can be obtained with the Forsus FRD in conjunction with comprehensive orthodontic treatment.

## Background

A common practice to correct a class II malocclusion with a retrognathic mandible is to use a functional appliance [1-4]. The early functional appliances are removable in nature and dependent on patient compliance for effectiveness. Removable appliances, such as the Frankel regulator, Bionator (Wehrheim, Germany), Activator (LM-Instruments Oy, Parainen, Finland), Twin Block, and even class II elastics, often have inconsistent results due to the fact that these appliances require high levels of patient cooperation [5]. A major advantage of fixed functional devices such as the Herbst, Jasper Jumper (3M), MARA, and Forsus Fatigue Resistant Device (FRD; 3M Unitek, Monrovia, CA, USA) is that they are fixed and effective 24 h a day with minimal patient compliance. The treatment effects of the various types of Herbst appliances have been well documented in the literature. There is a significant amount of evidence that class II malocclusion with mandibular retrusion can be corrected with a combination of maxillary restraint, mandibular lengthening, dental changes, and glenoid fossa remodeling [3,6-8]. However, most of the earlier design of the Herbst appliance cannot be used in combination with multibracket therapy to reduce treatment time. The FRD was introduced in 1999 and claimed to have similar results as the Herbst appliance and Class II elastic treatment [9]. It was also recommended as an acceptable substitute for Class II elastics for non-compliant patients [10]. The original appliance consisted of a NiTi spring bar with a transparent plastic coating. The spring can be attached, via its bent ends, to bands and archwires of the fixed orthodontic appliance [11]. The FRD is a more refined appliance to eliminate the limited movement of the other class II correctors and also the fatigue factor. The compression spring has a three-part telescoping assembly to allow enough freedom of jaw opening. The issue of fatigue fracture was addressed in the spring design based on engineering principles. Several studies have evaluated the dental and skeletal short-term effects of comprehensive fixed appliance treatment combined with the FRD in class II patients. Few studies have compared with a control sample to evaluate the exact mechanism of the appliance [10-16]. The objective of this study was to investigate the cephalometric changes of 24 patients treated with FRD in conjunction with comprehensive orthodontic treatment and compared to untreated subjects who were matched in age, sex, and craniofacial morphology.

## Methods

This is a retrospective study composed of 56 consecutively treated patients treated with the Forsus FRD in conjunction with fixed orthodontic appliances from the office of one of the authors (I.S.). The study received approval from the Institutional Review Board of West Virginia University (H-21973). The approval was also granted from one of the authors (I.S.) for the use of orthodontic records from his office. The following inclusion criteria was used to obtain the sample: Patients in the late mixed or early permanent dentition with class II division 1 malocclusion who required FRD and comprehensive non-extraction orthodontic treatment with fixed appliances; no history of orthodontic treatment before the initial radiograph, acceptable quality radiographs for both time points, and remaining growth potential as confirmed by Cervical Vertebral Maturation index (CVM). Exclusion criteria included poor quality radiographs, missing radiographs for either time point, or no remaining growth potential as confirmed by CVM. The final sample size consisted of 24 patients (9 females and 15 males). The control group consisted of subjects from the Bolton-Brush study with no history of orthodontic treatment and was matched in age, sex, and craniofacial morphology with the experimental subjects.

The initial lateral cephalometric radiographs of the treatment subjects (*n* = 24) were taken 1 year prior to the start of treatment (T1) with an average age of 10.7 ± 1.5 years. The post-treatment lateral cephalometric radiographs were taken on an average 2.7 months after removal of fixed and Forsus appliances (T2) with an average age of 14.5 ± 1.2 years. Therefore, the average treatment time was 27.8 months, and the average time between the two radiographs was 3.8 years. The CVM for each patient was determined in the manner as described by Baccetti et al. [17]. The average CVM stage at T1 was 1.8. The average CVM stage at T2 was 4.9. This means that the initial pretreatment radiograph was taken before the peak of the pubertal growth spurt which is associated with the CVM stage 3, and the final radiograph was taken after the peak of the pubertal growth spurt. Therefore, the treatment group entered their peak pubertal growth spurt between the T1 and T2 radiographs.

For the control subjects (*n* = 24), the first radiograph from the Bolton Brush Study (t1) was taken at an average age of 10.3 ± 1.1 years, and the second radiograph was taken at 14.7 ± 1.5 years. No significant differences were found between the treatment and control groups for any of the time periods.

### Pretreatment craniofacial morphology

The pretreatment craniofacial morphology of the treated and control groups was compared to determine if any statistically significant differences were present before treatment with the Forsus FRD and fixed orthodontic appliances (Table 1). Seven out of 29 variables were found to be significantly different between the two groups. The wits, overjet, and molar relationship were larger in the treated as compared to the control group, indicating that the treated group had a more severe class II anterior-posterior skeletal discrepancy than the control group. The Is-NL and overbite were also larger, and Mic-ML was smaller in the treated group, indicating that the treated group had a deeper overbite with more maxillary incisor eruption and less mandibular molar eruption. Overall, the pretreatment morphology between the treatment and control groups were quite similar.

**Table 1:** **Calculation of overjet and molar relationship changes**

**Overjet**	**Molar relationship**
Skeletal contributions	Skeletal contributions
1. OLp-Apt	1. OLp-Apt
2. OLp-Pg	2. OLp-Pg
Dental contributions	Dental contributions
3. Is-OLp minus OLp-Apt	3. Ms-OLp minus OLp-Apt
4. Ii-OLp minus OLp-Pg	4. Mi-OLp minus OLp-Pg
Overjet correction	Molar relationship correction
Sum of 1, 2, 3, and 4	Sum of 1, 2, 3, and 4

### Appliances for class II correction

**Figure 1:**
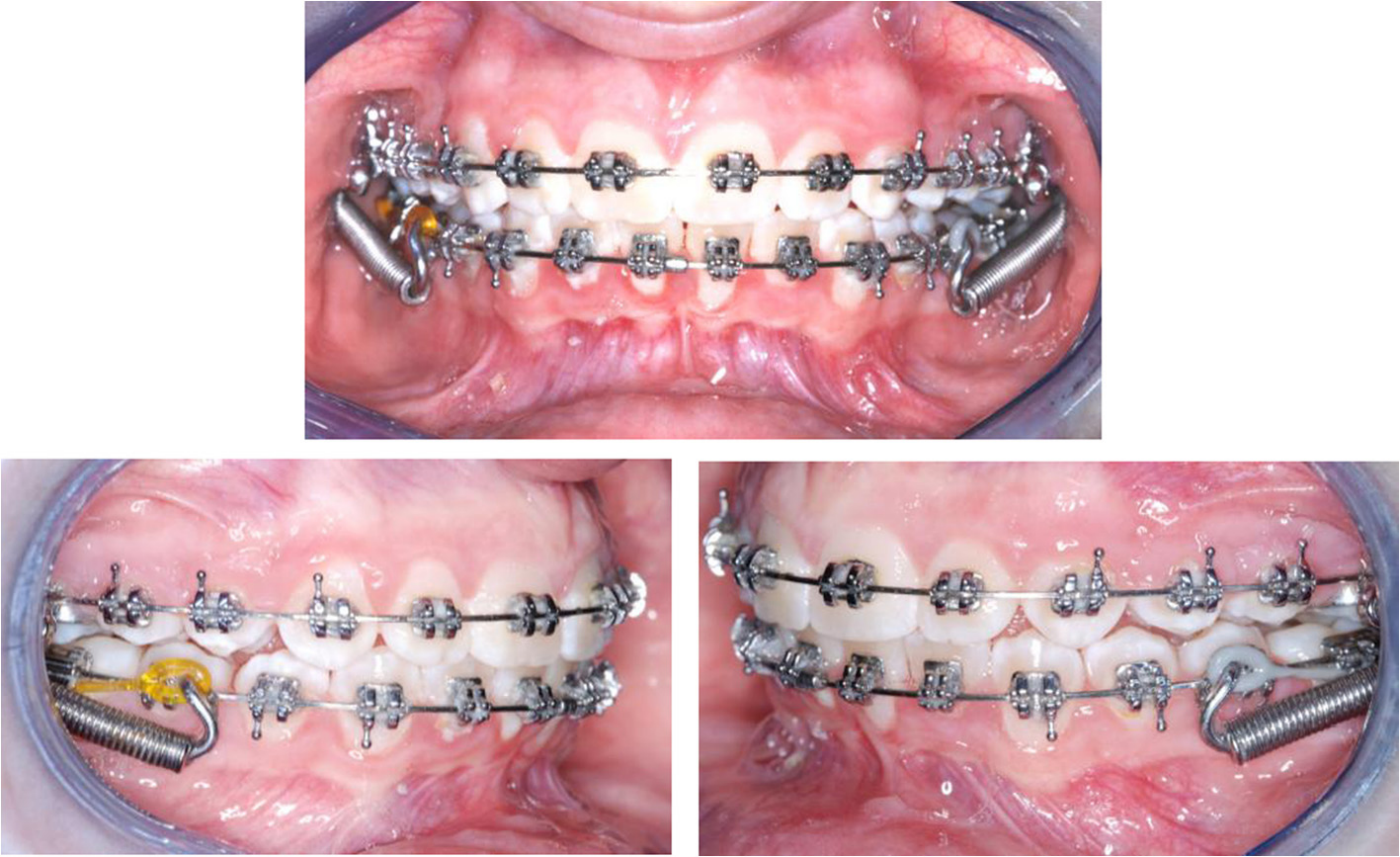
**Forsus Fatigue Resistant device with the three-piece telescoping coaxial spring.** The spring attaches to the buccally offset occlusal headgear tube on the maxillary molar, and a direct push rod attaches directly on the mandibular archwire.

The upper and lower molars were banded with Unitek 0.022 slot MBT prescription bands. The upper first molar bands had an occlusal headgear tube which allowed the engagement clip of the pushrod device to secure to it. The upper and lower second premolars to the second premolars were bonded using the Unitek Victory Series 0.022 slot Low Profile MBT brackets. The lower incisor brackets had a −6° inclination to help minimize the anterior proclination of the incisors which was a side effect of class II correction. The teeth were leveled and aligned using an archwire sequence of 0.014 NiTi, 16 × 22 NiTi, 16 × 22 stainless steel (SS), and 19 × 25 SS. The FRD appliance was placed once the upper and lower arches were leveled and aligned and a 0.019 × 0.025 SS wire was in place. The Forsus FRD with EZ clip was attached to the occlusal headgear tube on the upper first molar and the lower 19 × 25 SS wire between the lower first premolar and the lower canine. The maxillary and mandibular arches were colligated from the first molar to the contralateral first molar on a 19 × 25 SS wire to minimize any unwanted proclination of the lower incisors, as per manufacturer's instructions. The Forsus FRD was then left in place for between 6 to 12 months with an average time of 9 months, depending on the severity of the malocclusion. Overcorrection with the Forsus FRD was achieved where possible to account for relapse. After Forsus FRD removal, the occlusion was finalized using the same 19 × 25 SS wires and then all orthodontic appliances were removed. Once all the appliances were removed, the upper and lower teeth are retained with upper and lower Hawley retainers (Figure 1).

### Cephalometric analysis

**Figure 2:**
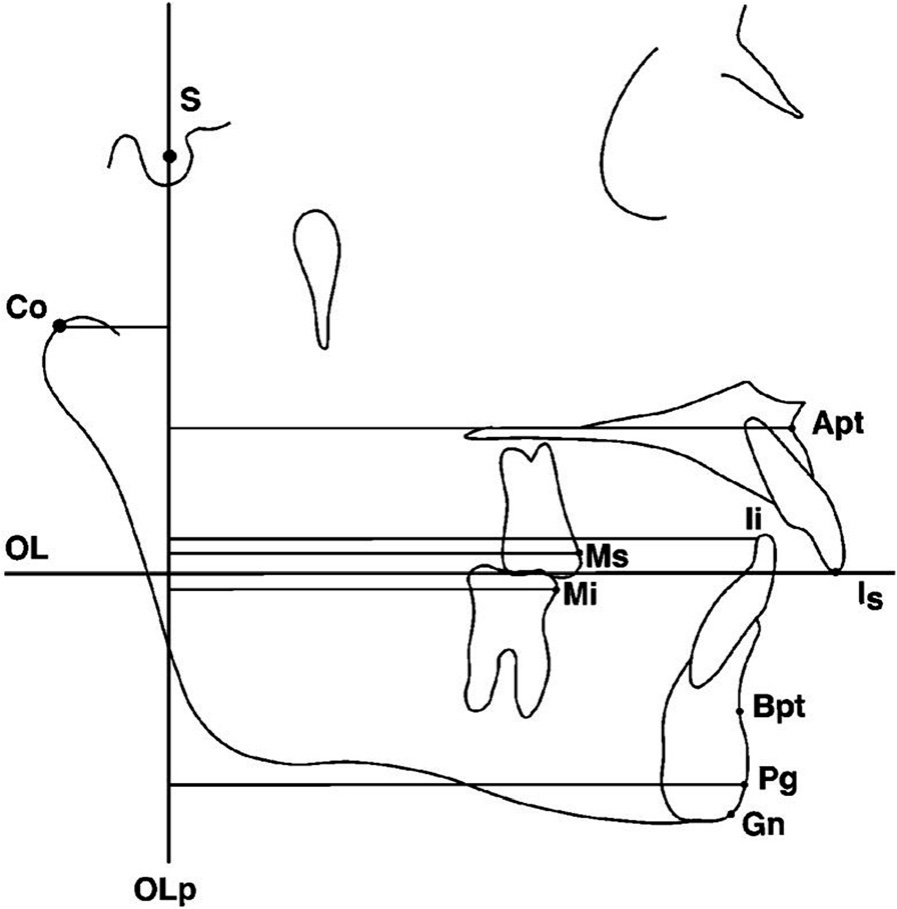
**Cephalometric landmarks and lines for sagittal measurements.**

**Figure 3:**
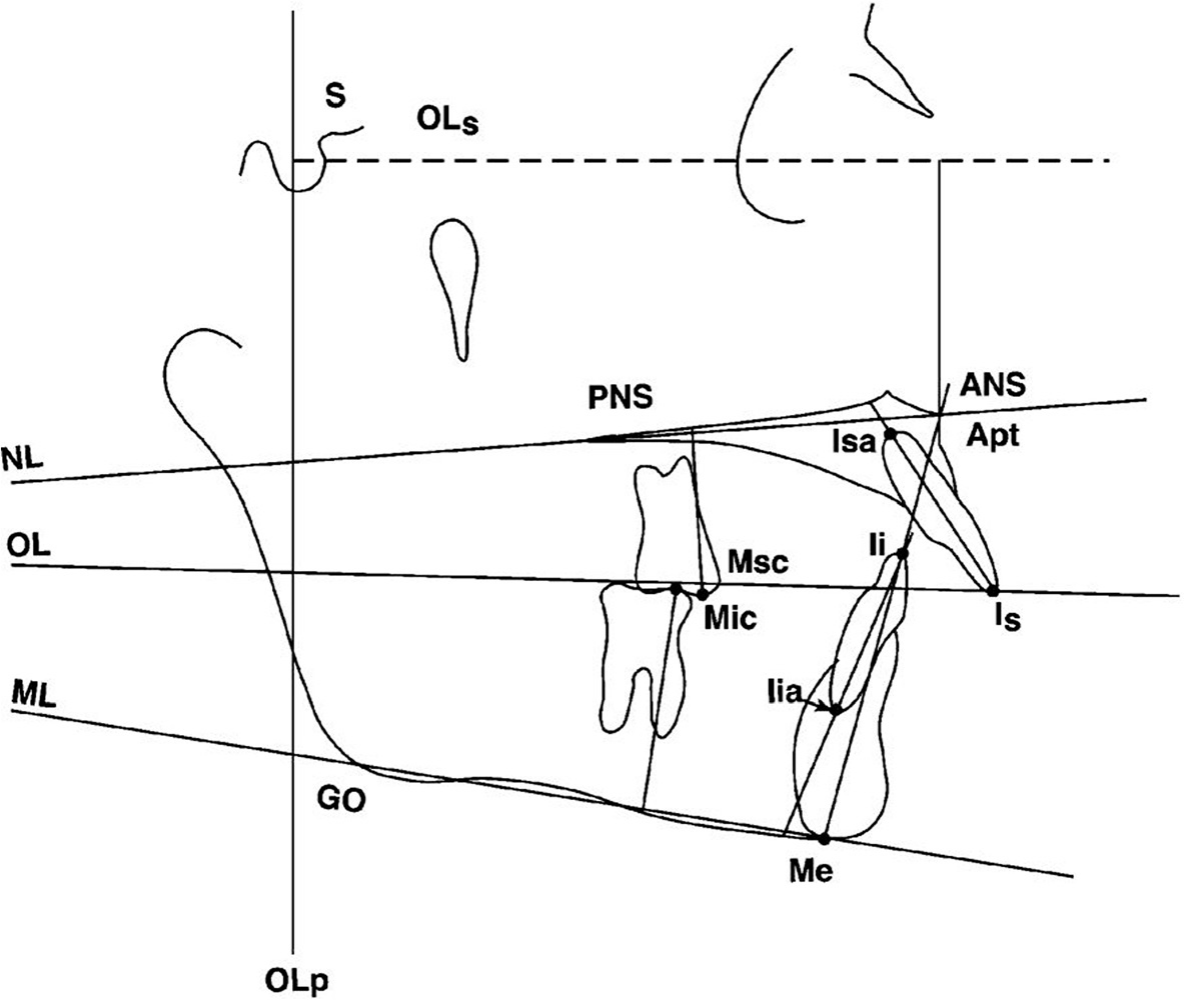
**Cephalometric landmarks and lines for vertical measurements.**

**Figure 4:**
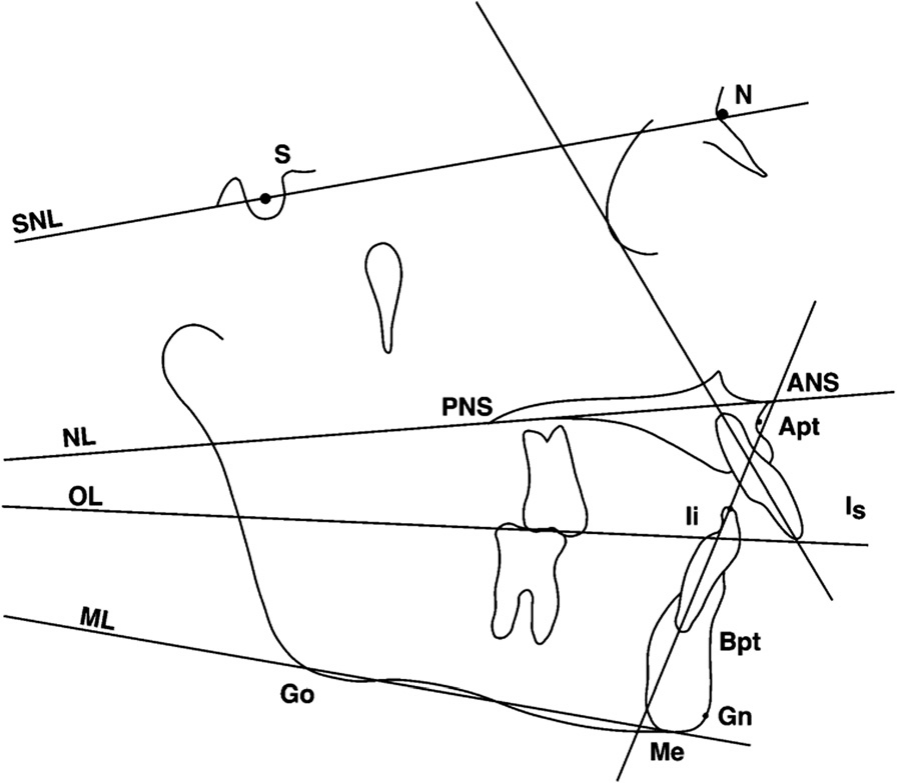
**Cephalometric landmarks and lines for angular measurements.**

Tracings were performed by one operator using a #2 HB mechanical lead pencil (Zebra 0.5-mm lead, Edison, NJ, USA), an orthodontic protractor (3M Unitek), and 0.003-in matte cephalometric acetate tracing film (3M Unitek). A custom cephalometric analysis was performed as described by Gunay et al. [14], Bjork [18], and Pancherz [19-20]. The landmarks used are defined in Figures 2,3,4. The measurement for each angular variable was performed by using a cephalometric protractor and was measured to the nearest 0.5°. The measurement for each sagittal and vertical measurement was performed with an electronic digital caliper (S225, Fowler, Boston, MA, USA) and measured to the nearest 0.1 mm. Analysis of the sagittal skeletal and dental changes was recorded along the occlusal plane (OLs) and to the occlusal plane perpendicular (OLp) from the first cephalogram, which formed the reference grid. The grid was then transferred to subsequent cephalograms by superimposing on the mid-sagittal cranial structure.

### Calculation of overjet and molar relationship correction

To determine the amount of skeletal and dental contribution to the overjet and molar relationship correction, the amount of dental change in the maxilla and mandible was calculated as shown in Table 2.

**Table 2:** **Correlation coefficients for all variables at T1 and T2**

**Variables**	**T1**			**T2**		
	**Mean (first)**	**Mean (second)**	**Correlation**	**Mean (first)**	**Mean (second)**	**Correlation**
Sagittal						
1. OLp-Apt	70.3	70.6	0.99	74.1	73.8	0.97
2. OLp-Pg	71.1	71.2	0.98	77.3	77.3	0.99
3. OLp-Co	10.5	10.2	0.99	12.3	12.6	0.98
4. Co-Apt	81.3	81.4	0.98	87.1	86.9	0.98
5. Co-Gn	98.5	98.1	0.99	108.6	108.9	0.99
6. Co-Gn minus Co-Apt	17.1	16.7	0.97	21.5	22.0	0.99
7. Wits	1.5	1.6	0.97	−0.3	−0.3	0.98
8. Is-OLp	78.4	78.6	0.99	80.9	80.9	0.99
9. Ii-OLp	70.8	71.0	0.99	77.5	77.4	0.98
10. Overjet	7.7	7.6	0.99	3.4	3.5	0.89
11. Ms-OLp	48.1	48.1	0.99	52.9	53.0	0.98
12. Mi-OLp	47.4	48.0	0.95	55.4	54.8	0.98
13. Molar relationship	0.8	0.1	0.90	−2.5	−1.8	0.79
Vertical						
14. OLs-Apt	29.1	28.7	0.99	32.5	32.4	0.98
15. ANS-Me	58.0	57.8	0.99	62.9	62.9	0.99
16. Is-NL	29.0	29.1	0.98	30.3	30.5	0.99
17. Ii-ML	36.2	36.2	0.99	37.7	37.9	0.99
18. Overbite	3.9	3.9	0.98	2.2	2.3	0.98
19. Msc-NL	18.7	18.6	0.98	21.7	21.6	0.99
20. Mic-ML	25.2	25.4	0.98	29.0	29.2	0.98
Angular						
21. SNA	81.6	82.0	0.97	81.5	81.3	0.98
22. SNB	76.1	76.3	0.98	77.1	77.2	0.99
23. ANB	5.6	5.7	0.95	4.4	4.1	0.96
24. SNL-NL	6.9	6.5	0.96	7.0	7.2	0.98
25. SNL-ML	34.0	34.3	0.99	33.3	33.1	0.99
26. SNL-OL	20.3	20.6	0.97	19.5	19.7	0.97
27. Is/NL	115.6	115.4	0.98	112.3	112.9	0.98
28. Ii/ML	92.2	91.5	0.99	96.3	95.8	0.99
29. Inter-incisal angle	124.2	124.8	0.99	124.7	124.8	0.99

### Data analysis

Normal quantile plots were used to check the assumption of normality. A matched paired *t* test was performed for each variable to identify the overall treatment effects of the fixed orthodontic appliances and the Forsus FRD appliance (Tx (T2 − T1)) minus (control (t2 − t1)). A level of significance of 0.05 was used in this study.

### Error study

For the cephalometric analysis, the error in locating, superimposing, and measuring the changes of the different landmarks by one examiner (intra-examiner error) was performed on cephalograms of ten randomly selected subjects. All cephalograms were recorded twice independently on two separate occasions with a 2-week interval between. For all cephalometric variables, the differences between the measurements from the first and second tracings were compared for each individual at T1 and T2. A matched paired *t* test was performed to compare the two sets of measurements. A correlation coefficient was established for each variable at each time point to determine the degree of reliability (Table 3). Results show that the method of cephalometric analysis used in this study was determined to be reliable. This included the identification of landmarks, superimposition of radiographs, and the measurements taken at each time point. The correlations ranged from 0.79 to 0.99, which means that the method of data collection was reliable.

**Table 3:** **Comparison of the pretreatment craniofacial morphology in pooled subjects**

	**Pooled (males and females)**						
**Variable**	**Control**		**Treated**		***p*** **value**		**Significance**
	**Mean**	**SD**	**Mean**	**SD**		**Difference**	
Sagittal							
OLp-Apt	70.3	4.2	70.9	4.5	0.6684	0.6	NS
OLp-Pg	73.9	4.8	72.4	5.3	0.3286	−1.5	NS
OLp-Co	10.0	2.2	9.8	3.4	0.7782	−0.2	NS
Co-Apt	80.7	4.1	81.3	5.3	0.6852	0.6	NS
Co-Gn	99.7	5.3	99.3	7.0	0.8091	−0.5	NS
Co-Gn minus Co-Apt	19.0	3.0	18.0	3.5	0.2948	−1.0	NS
Wits	0.6	1.7	1.8	1.9	0.0269	1.2	*
Is-OLp	77.2	5.5	78.9	5.4	0.2925	1.7	NS
Ii-OLp	71.9	4.8	71.1	4.8	0.5618	−0.8	NS
Overjet	5.3	1.6	7.8	2.9	0.0008	2.5	*
Ms-OLp	49.2	3.8	48.8	4.7	0.7440	−0.4	NS
Mi-OLp	49.0	4.3	47.5	4.6	0.2549	−1.5	NS
Molar relationship	0.2	0.9	1.3	1.8	0.0117	1.1	*
Vertical							
OLs-Apt	26.9	2.3	30.2	5.7	0.0131	3.3	*
ANS-Me	59.0	5.1	57.5	5.8	0.3620	−1.5	NS
Is-NL	26.0	2.6	28.5	3.1	0.0036	2.6	*
Ii-ML	35.4	3.3	36.3	3.3	0.3720	0.9	NS
Overbite	3.1	1.4	3.9	1.2	0.0255	0.8	*
Msc-NL	18.5	2.0	18.6	2.1	0.8809	0.1	NS
Mic-ML	27.0	2.4	25.5	2.4	0.0449	−1.5	*
Angular							
SNA	80.1	2.8	81.1	3.1	0.2623	1.0	NS
SNB	75.8	2.9	75.9	2.6	0.8411	0.2	NS
ANB	4.3	1.3	5.1	1.8	0.0796	0.8	NS
SNL-NL	6.4	2.9	7.2	3.0	0.3755	0.8	NS
SNL-ML	33.4	4.8	32.8	5.9	0.6801	−0.7	NS
SNL-Olf	19.9	3.1	18.8	3.7	0.2901	−1.1	NS
Is/NL	110.1	5.0	110.9	22.4	0.8837	0.7	NS
Ii/ML	95.1	6.0	93.7	6.7	0.4385	−1.5	NS
Inter-incisal angle	127.5	7.5	125.2	11.7	0.4427	−2.3	NS

* = p<.05.

## Results

### Comparison of changes in the treated group (T2 − T1) vs. the control group (t2 − t1)

Treatment effects of the FRD were calculated by subtracting growth changes (t2 − t1) from treatment changes (T2 − T1). A total of 29 sagittal, vertical, and angular variables were evaluated for each group. Tables 4 and 5 compare the changes in the treatment group (T2 − T1) vs. the control group (t2 − t1) in the male and female subjects, respectively. Gender differences were found in six variables (Go-Gn minus Co-Apt; Is-OLp, Ii-ML, SNA, SNL-Olf, and Ii/ML).

**Table 4:** **Comparison of changes in the treatment group (T2 − T1) vs. control group (t2 − t1) in the male subjects**

	**Males**						
**Variable**	**Control (t2 − t1)**		**Treated (T2 − T1)**		***p*** **value**		**Significance**
	**Mean**	**SD**	**Mean**	**SD**		**Difference**	
Sagittal							
OLp-Apt	6.4	2.4	4.2	2.7	0.0814	−2.2	NS
OLp-Pg	7.8	3.9	8.0	4.2	0.9110	0.2	NS
OLp-Co	0.2	1.9	1.8	2.0	0.1034	1.5	NS
Co-Apt	6.6	3.1	5.9	2.8	0.6072	−0.7	NS
Co-Gn	9.9	4.2	11.7	3.6	0.3041	1.8	NS
Co-Gn minus Co-Apt	3.3	1.6	5.9	2.2	0.0148	2.5	*
Wits	0.3	1.4	−2.0	2.1	0.0149	−2.3	*
Is-OLp	7.0	3.3	3.4	4.9	0.0901	−3.6	NS
Ii-OLp	7.1	2.8	8.0	2.9	0.5245	0.8	NS
Overjet	−0.1	1.0	−4.6	3.3	0.0024	−4.5	*
Ms-OLp	7.5	4.1	5.3	3.4	0.2058	−2.2	NS
Mi-OLp	7.6	4.0	9.4	3.6	0.3090	1.8	NS
Molar relationship	−0.1	1.2	−4.1	2.2	0.0003	−4.0	*
Vertical							
Ols-Apt	3.6	1.3	3.0	2.0	0.4281	−0.7	NS
ANS-Me	5.6	2.7	6.4	3.2	0.5730	0.8	NS
Is-NL	1.9	1.2	1.4	2.0	0.5925	−0.4	NS
Ii-ML	4.0	1.4	2.6	3.1	0.2563	−1.5	NS
Overbite	0.6	1.9	−1.7	1.2	0.0029	−2.3	*
Msc-NL	3.0	1.2	3.2	1.8	0.7906	0.2	NS
Mic-ML	3.3	1.7	4.6	2.1	0.1870	1.3	NS
Angular							
SNA	2.0	1.4	0.2	1.3	0.0075	−1.8	*
SNB	1.5	1.5	1.8	1.5	0.7032	0.3	NS
ANB	0.5	0.4	−1.6	1.3	0.0005	−2.1	*
SNL-NL	0.5	1.6	−0.8	1.3	0.0523	−1.3	NS
SNL-ML	−0.4	2.0	−1.9	3.0	0.2593	−1.4	NS
SNL-Olf	−1.1	3.9	−1.4	2.2	0.7791	−0.4	NS
Is/NL	−0.8	3.7	4.0	26.9	0.6504	4.8	NS
Ii/ML	−0.4	3.3	3.7	5.1	0.0622	4.2	NS
Inter-incisal angle	2.4	6.5	−0.2	12.5	0.6054	−2.7	NS

* = p<.05.

**Table 5:** **Comparison of treatment group (T2 − T1) vs. control group (t2 − t1) in the female subjects**

	**Females**						
**Variable**	**Control (T2 − T1)**		**Treated (T2 − T1)**		***p*** **value**		**Significance**
	**Mean**	**SD**	**Mean**	**SD**		**Difference**	
Sagittal							
OLp-Apt	4.5	2.4	3.5	2.0	0.2924	−1.0	NS
OLp-Pg	6.1	2.6	6.2	3.2	0.9615	0.1	NS
OLp-Co	1.4	1.6	1.0	2.1	0.6330	−0.4	NS
Co-Apt	6.0	3.4	4.6	3.8	0.3796	−1.4	NS
Co-Gn	9.0	3.5	8.2	4.2	0.6021	−0.8	NS
Co-Gn minus Co-Apt	3.1	2.5	3.6	1.8	0.5992	0.5	NS
Wits	0.6	1.5	−2.6	1.6	0.0001	−3.2	*
Is-OLp	4.9	2.1	2.2	4.1	0.0452	−2.7	*
Ii-OLp	4.8	2.1	6.7	3.1	0.0897	1.9	NS
Overjet	0.1	1.5	−4.4	2.2	0.0001	−4.6	*
Ms-OLp	5.9	1.9	3.9	3.7	0.0933	−2.0	NS
Mi-OLp	6.7	2.4	8.2	3.8	0.2419	1.5	NS
Molar relationship	−0.7	1.1	−4.2	1.4	0.0001	−3.5	*
Vertical							
OLs-Apt	2.9	1.9	2.6	1.8	0.7111	−0.3	NS
ANS-Me	4.4	2.4	2.8	3.1	0.1785	−1.6	NS
Is-NL	1.5	1.7	0.8	1.8	0.3345	−0.7	NS
Ii-ML	2.8	1.5	0.5	2.6	0.0143	−2.2	*
Overbite	0.0	1.1	−2.0	0.9	0.0002	−2.0	*
Msc-NL	3.0	1.7	1.6	1.3	0.0537	−1.3	NS
Mic-ML	2.3	1.5	3.1	1.4	0.2109	0.8	NS
Angular							
SNA	1.4	1.9	0.1	0.9	0.0549	−1.4	NS
SNB	1.4	1.3	1.5	1.0	0.7948	0.1	NS
ANB	0.1	1.4	−1.4	1.0	0.0079	−1.5	*
SNL-NL	0.1	1.9	0.2	2.7	0.8965	0.1	NS
SNL-ML	−0.8	1.0	−1.1	1.9	0.6728	−0.3	NS
SNL-Olf	−2.4	2.7	0.3	2.0	0.0152	2.7	*
Is/NL	0.2	2.8	0.8	11.1	0.8564	0.5	NS
Ii/ML	−0.6	3.6	4.2	3.9	0.0053	4.9	*
Inter-incisal angle	1.4	5.6	−3.1	10.0	0.1722	−4.5	NS

* = p<.05.

Table 4 shows the comparison of changes in the treatment group (T2 − T1) vs. the control group (t2 − t1) in the pooled subjects. Significant differences were found in 12 out of the 29 variables. The position of the maxillary base (OLp-Apt) came forward 3.9 mm in the treated group and 5.1 mm in the control group, resulting in a net 1.2 mm of restricted forward movement of the maxillary base (*p* < 0.1) by the FRD appliance. During 3.8 years of treatment, the position of the mandibular base (OLp-Pg) came forward 7.3 mm in the treated group and 6.6 mm in the control, resulting in a net forward movement of 0.7 mm by the appliance (*p* < 0.5). The difference between the effective maxillary and mandibular length (Co-Gn minus Co-Apt) was found to be significantly different between the treatment and control groups (1.9 mm, *p* < 0.009). The position of the maxilla relative to the mandible along the functional occlusal plane (Wits) showed a significant difference of −2.7 mm (*p* < 0.001). The position of the maxillary incisor (Is-OLp) came back −1.5 mm with the appliance (*p* < 0.02), and the position of the mandibular incisors (Ii-OLp) came forward 1.3 mm after subtracting the growth (*p* < 0.02). The overjet correction was corrected 4.6 mm after subtracting the growth (*p* < 0.0001). The maxillary molar (Ms-OLp) moved back 0.5 mm with the appliance (*p* < 0.09), and the mandibular molar (Mi-OLp) came forward 1.3 mm after subtracting the growth (*p* < 0.04). The molar relationship was corrected 3.6 mm after subtracting changes due to growth (*p* < 0.0001). Similar findings were observed with the male and female subjects that were analyzed separately (Tables 5 and 6).

**Table 6:** **Comparison of changes in the treatment group (T2 − T1) vs. control group (t2 − t1) in the pooled subjects**

	**Pooled (males and females)**						
**Variable**	**Control (t2 − t1)**		**Treated (T2 − T1)**		***p*** **value**		**Significance**
	**Mean**	**SD**	**Mean**	**SD**		**Difference**	
Sagittal							
OLp-Apt	5.1	2.5	3.9	2.4	0.1148	−1.2	NS
OLp-Pg	6.6	3.1	7.3	3.9	0.5216	0.7	NS
OLp-Co	1.0	1.7	1.5	2.0	0.4038	0.5	NS
Co-Apt	6.2	3.3	5.4	3.2	0.4360	−0.8	NS
Co-Gn	9.3	3.7	10.4	4.1	0.3453	1.1	NS
Co-Gn minus Co-Apt	3.1	2.2	5.0	2.3	0.0086	1.9	*
Wits	0.5	1.5	−2.2	1.9	0.0001	−2.7	*
Is-OLp	5.6	2.7	2.9	4.6	0.0218	−2.6	*
Ii-OLp	5.5	2.5	7.5	3.0	0.0212	2.0	*
Overjet	0.1	1.3	−4.5	2.9	0.0001	−4.6	*
Ms-OLp	6.4	2.8	4.8	3.5	0.0928	−1.6	NS
Mi-OLp	7.0	2.9	8.9	3.6	0.0495	2.0	*
Molar relationship	−0.5	1.2	−4.1	1.9	0.0001	−3.6	*
Vertical							
Ols-Apt	3.2	1.7	2.8	1.9	0.5570	−0.3	NS
ANS-Me	4.8	2.5	5.1	3.6	0.7537	0.3	NS
Is-NL	1.6	1.6	1.2	1.9	0.4031	−0.4	NS
Ii-ML	3.2	1.6	1.8	3.0	0.0680	−1.4	NS
Overbite	0.2	1.4	−1.8	1.1	0.0001	−2.0	*
Msc-NL	3.0	1.5	2.6	1.8	0.4641	−0.4	NS
Mic-ML	2.6	1.6	4.0	2.0	0.0126	1.4	*
Angular							
SNA	1.6	1.8	0.1	1.2	0.0013	−1.5	*
SNB	1.4	1.3	1.7	1.3	0.5147	0.3	NS
ANB	0.2	1.2	−1.5	1.2	0.0001	−1.8	*
SNL-NL	0.2	1.8	−0.4	1.9	0.2456	−0.6	NS
SNL-ML	−0.7	1.4	−1.6	2.6	0.1634	−0.9	NS
SNL-Olf	−2.0	3.1	−0.8	2.2	0.1382	1.2	NS
Is/NL	−0.1	3.1	2.8	22.0	0.5489	2.9	NS
Ii/ML	−0.6	3.4	3.9	4.6	0.0005	4.5	*
Inter-incisal angle	1.7	5.8	−1.3	11.5	0.2744	−3.0	NS

For vertical changes, the overbite was decrease by 2.0 mm more in the treatment relative to the control group (*p* < 0.0001), and the lower molar (Mic-ML) in the treatment group erupted 1.4 mm more in the treatment relative to the control group (*p* < 0.01). Similar findings were observed with the male and female subjects that were analyzed separately (Tables 5 and 6).

For angular changes, SNA was decreased 1.5° more in the treatment relative to the control group (*p* < 0.001), and ANB was decreased 1.8° more in the treatment relative to the control group (*p* < 0.0001). The inclination of the mandibular incisor (Ii/ML) was increased 4.5° more in the treatment relative to the control group (*p* < 0.0005). Similar findings were observed with the male and female subjects that were analyzed separately (Tables 5 and 6).

### Contributions to net overjet and net molar relationship corrections

**Figure 5:**
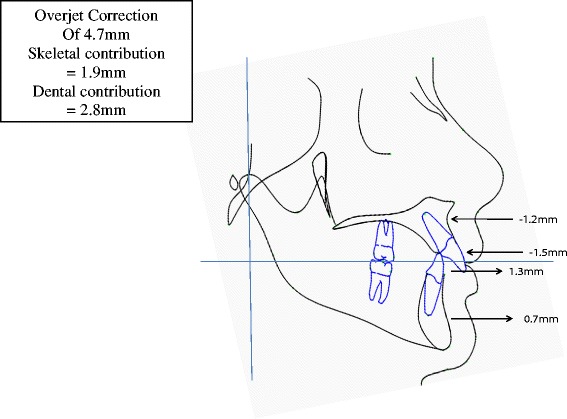
**Components of net overjet correction (T2 − T1).**

**Figure 6:**
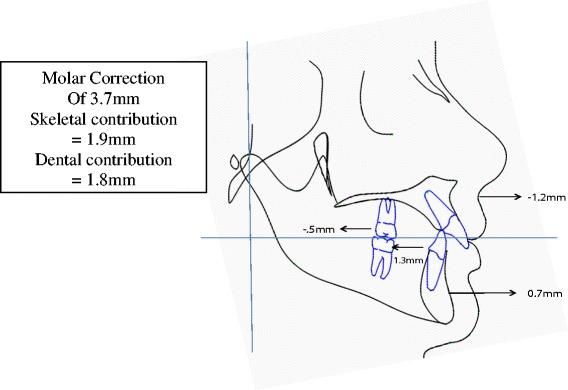
**Components of net molar correction (T2 − T1).**

**Figure 7:**
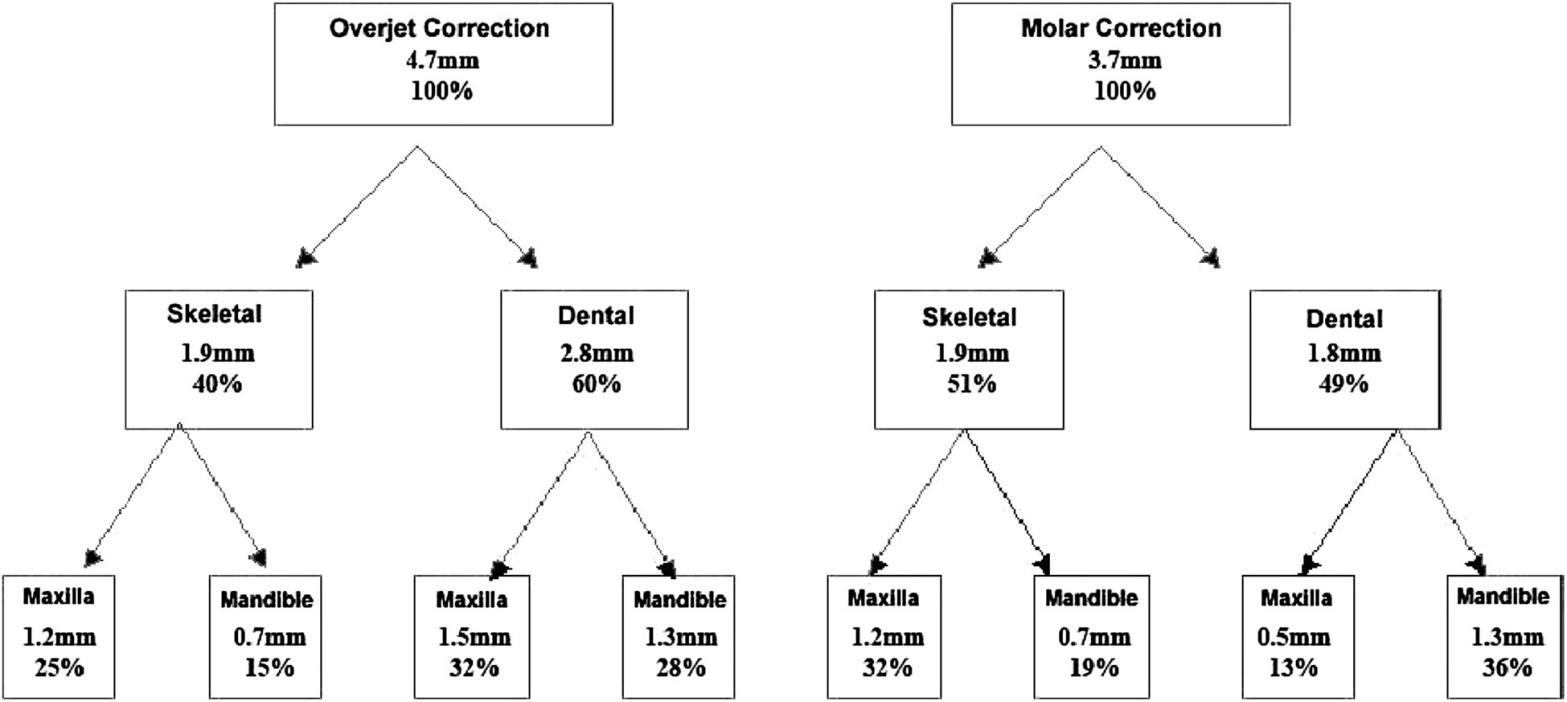
**Pitchfork analysis of net overjet and molar relationship (T2 − T1).**

Figure 5 shows the net overjet correction after subtracting growth from treatment changes. The overjet was improved by an average of 4.7 mm. Of the correction, 1.9 mm (40%) was due to skeletal changes, and 2.8 mm (60%) of the correction was due to dental changes. This was a result of 1.2 mm of restraint in forward maxillary growth, 0.7 mm of forward movement of the mandible, 1.5 mm of backward movement of the maxillary incisors, and 1.3 mm forward movement of the mandibular incisors. Figure 6 shows the net molar relationship correction. The molar relationship was corrected by an average of 3.7 mm. Of the correction, 1.9 mm (51%) was skeletal in nature, and 1.8 mm (49%) of the correction was dental in nature. This was a result of the skeletal changes above and 0.5 mm of backward movement of the maxillary molars and 1.3 mm of forward movement of the mandibular molars. Figure 7 shows the pitchfork analysis diagram to describe the net skeletal and dental contributions related to the overjet and molar relationship corrections.

## Discussion

In the present study, 24 patients were treated consecutively, utilizing identical appliance and force system by the same operator. A limitation of this study is that the sample was collected retrospectively. The methodology used increase the potential selection and proficiency biases of the study. The extra effort in matching the treatment group with an untreated control sample that was matched in age and craniofacial morphology allows growth to be subtracted from treatment changes in order to obtain the treatment effect of the appliance. However, the severity of class II malocclusion was not included in the criteria of selecting subjects. The average ANB of the treatment subjects was 5.1° ± 1.8° compared to the average ANB of the control subjects from the Bolton Brush Study which was 4.3° ± 1.3° (Table 1).

Registration of the cephalograms was undertaken by the same examiner in order to reduce method error. The reference grid used in the evaluation of the sagittal changes made it possible to evaluate the skeletal and dental changes that occurred in the maxilla and mandible along the occlusal plane (OLs). Since all before and after treatment sagittal measurements were made with reference to the same reference plane (before treatment occlusal plane perpendicular (OLp), downward and backward rotation of the occlusal plane (OLs) which occurred during treatment would not affect the reference grid and bias the results.

In this study, the net overjet correction with an average treatment time of 27.8 months was 4.7 mm. Heinig and Goz found a 4.7-mm reduction in overjet with the Forsus Flat Spring (3M Unitek) [12]. Karacay et al. found a 3.7-mm reduction in net overjet with the Forsus NiTi Flat Spring relative to an untreated control group [11]. Jones et al. found a 3.2-mm reduction in overjet with the Forsus FRD for 2.7 years of treatment, and the changes were similar to treatment with intermaxillary elastics with a 2.8-mm reduction in overjet [10]. Franchi et al. reported a 5.5-mm reduction in net overjet relative to an untreated control group [13]. These results are also similar to those reported on the Herbst appliance with a net overjet correction ranging from 2.7 to 5.2 mm [21-25]. The change in overjet was partially contributed by a restraint in the forward movement of the maxilla by 1.2 mm. Franchi et al. also reported a restraint in sagittal growth of 1.2 mm for ‘A’ point to Nasion perp [13]. Karacay et al. found a net posterior movement of ‘A’ point of 0.7 mm with the Forsus Flat Spring [11]. The slightly more posterior movement of the ‘A’ point in this study is probably due to the longer period of time that the Forsus FRD was left in place (average 9 months). This headgear effect has also been reported by fixed functional appliances such as the Herbst appliance [21-25], Jasper Jumper therapy [26,27]_,_ and intermaxillary elastics [10]. The mandibular base contributed 0.7 mm for the change in overjet correction. Franchi et al. reported a 1.8-mm increase in the total mandibular length (Co-Gn) with respect to untreated controls [13]. Karacay et al. found a net forward movement of the mandibular base of 1.0 mm relative to a control group with the Forsus NiTi Flat Spring [11]. Other studies with the Herbst appliance reported a greater forward movement in the mandibular base ranging from 0.9 to 4.5 mm [2,21,22]. However, a controlled clinical trial [7] following patients treated with FRD for 2.3 years after completion of comprehensive treatment showed no significant sagittal changes but mainly dentoalveolar changes [14].

The overjet correction was also partially contributed by dentoalveolar changes. In this study, a retraction of the maxillary incisors (1.5 mm) and a forward movement of the mandibular incisors (1.3 mm) were noted. Karacay et al. found a 1.4-mm net retraction of the maxillary incisor and a 2.2-mm net protrusion of the lower incisor with the Forsus NiTi Flat Spring [15]. Franchi et al. reported significant retrusion of the upper incisors (1.5 mm) and proclination of the mandibular incisors (2.5 mm) [13]. This is probably a side effect of the telescoping coxial spring which has a tendency to procline the lower incisors similar to intermaxillary elastics.

The molar relationship was improved by a total of 3.6 mm. Heinig and Goz [12] and Jones et al. [10] found a molar relationship improvement of 3.9 and 3.2 mm, respectively, without a control group. Other studies with the Herbst appliance reported an improvement ranging from 2.4 to 4.6 mm [21-25]. The change in molar relationship was contributed by changes in apical base as described above as well as a distal movement of the maxillary molars (0.5 mm) and a mesial movement of the mandibular molars (1.3 mm). Heinig and Goz found a 1.1 mm of distal maxillary molar movement and 1.7 mm of mesial mandibular molar movement with the Forsus NiTi Flat Spring [12]. Karacay et al. found a 1.97 mm of distal maxillary molar movement and 1.75 mm of mesial mandibular molar movement compared to a control group [11]. Other studies with the Herbst appliance showed a range of 0.4 to 1.5 mm of distal maxillary molar movement and −0.3 to 1.6 mm of mesial mandibular molar movement [21-25]. However, a controlled clinical trial following patients treated with FRD for 2.3 years after completion of comprehensive treatment showed no significant sagittal changes but mainly dentoalveolar changes [14].

The overbite was found to decrease with treatment (2.0 mm). This was accompanied by a slight increase in lower facial height (0.3 mm). However, the palatal plane (SNL-NL, 0.6°), mandibular plane (SNL-ML, 0.9°), and occlusal plane (SNL-OLs, 1.2°) all showed changes which were not statistically significant. There was a net intrusion of the maxillary molar of 0.4 mm. This is in contrast to treatment of class II malocclusion with class II elastics that is usually accompanied by extrusion of the posterior molars [10]. Similar reductions in the palatal plane and mandibular plane were reported for the Forsus NiTi Flat Spring and Forsus FRD. Karacay et al. reported a net increase of 2.81° for the occlusal plane with the Forsus NiTi Flat Spring [11]. They also found a net intrusion of the maxillary molars by 3.9 mm when measured with reference to the SN plane. Heinig and Goz found a 4.2° increase in the occlusal plane, which was slightly larger than the increase found in this study [12]. This may be due to the fact that there are different definitions of where to measure the functional occlusal plane and may explain the differences between the studies. For the incisors, the maxillary incisor (Is-NL) was found to intrude by 0.4 mm and the mandibular incisor (Ii-NL) by 1.4 mm. Karacay et al. measured 3.1 mm of net mandibular incisor intrusion, which was more than that of the present study [11].

## Conclusions

Patients with mild to moderate class II malocclusion can be corrected with the Forsus FRD appliance in conjunction with comprehensive orthodontic treatment. The change in overjet and correction of molar relationship was attributed to a headgear effect on the maxilla together with a retraction of the maxillary incisors and mesial movement of the mandibular incisors. Unlike treatment with class II elastics, there was no excessive extrusion of the posterior molars and incisors.

## References

[1] Tulloch JF, Proffit WR, Phillip C (2004). Outcomes in a 2-phase randomized clinical trial of early Class II treatment. Am J Orthod Dentofacial Orthop.

[2] McNamara JA, Bookstein FL, Shaughnessy TG (1985). Skeletal and dental changes following functional regulator therapy on Class II patients. Am J Orthod Dentofacial Orthop.

[3] Pancherz HA (1984). Cephalometric analysis of skeletal and dental changes contributing to Class II correction in activator treatment. Am J Orthod Dentofacial Orthop.

[4] Falck F, Frankel R (1989). Clinical relevance of step-by-step mandibular advancement in the treatment of mandibular retrusion using the Frankel appliance. Am J Orthod Dentofacial Orthop.

[5] Sahm G, Bartsch A, Witt E (1990). Micro-electronic monitoring of functional appliance wear. Eur J Orthod.

[6] Pancherz H, Fischer S (2003). Amount and direction of temporomandibular joint growth changes in Herbst treatment: a cephalometric long-term investigation. Angle Orthod.

[7] Flores-Mir C, Ayeh A, Goswani A, Charkhandeh S (2007). Skeletal and dental changes in Class II division 1 malocclusions treated with splint-type Herbst appliances. A systematic review. Angle Orthod.

[8] Woodside DG, Metaxas A, Altuna G (1987). The influence of functional appliance therapy on glenoid fossa remodeling. Am J Orthod Dentofacial Orthop.

[9] Vogt W (2006). The Forsus Fatigue Resistant Device. J Clin Orthod.

[10] Jones G, Buschang PH, Kim KB, Oliver DR (2008). Class II non-extraction patients treated with the Forsus Fatigue Resistant Device versus intermaxillary elastics. Angle Orthod.

[11] Karacay S, Akin E, Olmez H, Gurton AU, Sagdic D (2006). Forsus Nitinol Flat Spring and Jasper Jumper corrections of Class II division 1 malocclusions. Angle Orthod.

[12] Heinig N, Goz G (2001). Clinical application and effects of the Forsus spring. A study of a new Herbst hybrid. J Orofac Orthop.

[13] Franchi L, Alvetro L, Giuntini V, Masucci C, Defraia E, Baccetti T (2011). Effectiveness of comprehensive fixed appliance treatment used with the Forsus Fatigue Resistant Device in Class II patients. Angle Orthod.

[14] Cacciatore G, Ghislanzoni LTH, Alvetro L, Giuntini V, Franchi L (2014). Treatment and posttreatment effects induced by the Forsus appliance: a controlled clinical study. Angle Orthod.

[15] Gunay EA, Arun T, Nalbantgil D (2011). Evaluation of the immediate dentofacial changes in late adolescent patients treated with the Forsus FRD. Eur J Dent.

[16] Aras A, Ada E, Saracoglu H, Gezer NS, Aras I (2011). Comparison of treatments with the Forsus Fatigue Resistant Device in relation to skeletal maturity: a cephalometric and magnetic resonance imaging study. Am J Orthod Dentofac Orthop.

[17] Baccetti T, Franch L, McNamara JA (2002). An improved version of the cervical vertebral maturation (CVM) method for the assessment of mandibular growth. Angle Orthod.

[18] Bjork A (1947). The Face in Profile: An Anthoropological X-ray Investigation of Swedish Children and Conscripts.

[19] Pancherz H (1982). The mechanism of Class II correction in Herbst appliance treatment. A cephalometric investigation. Am J Orthod Dentofacial Orthop.

[20] Pancherz H (1982). Vertical dentofacial changes during Herbst appliance treatment. A cephalometric investigation. Swed Dent J Suppl.

[21] VanLaecken R, Martin CA, Dischinger T, Razmus T, Ngan P (2006). Treatment effects of the edgewise Herbst appliance: a cephalometric and tomographic investigation. Am J Orthod Dentofacial Orthop.

[22] Croft RS, Buschang PH, English JD, Meyer R (1999). A cephalometric and tomographic evaluation of Herbst treatment in the mixed dentition. Am J Orthod Dentofacial Orthop.

[23] Hansen K, Pancherz H (1992). Long-term effects of Herbst treatment in relation to normal growth development: a cephalometric study. Eur J Orthod.

[24] Pancherz H, Hagg U (1985). Dentofacial orthopedics in relation to somatic maturation. An analysis of 70 consecutive cases treated with the Herbst appliance. Am J Orthod Dentofacial Orthop.

[25] Wigal T, Dischinger T, Martin CA, Razmus T, Gunel E, Ngan P (2011). Stability of Class II patients treated with the edgewise crown Herbst in the early mixed dentition. Am J Orthod Dentofacial Orthop.

[26] Cope JB, Buschang PH, Cope DD, Parker J, Blackwood HO (1994). Quantitative evaluation of craniofacial changes with Jasper Jumper therapy. Angle Orthod.

[27] Covell DA, Tremmell DW, Boero RP, West R (1999). A cephalometric study of Class II Division 1 malocclusions treated with the Jasper Jumper appliance. Angle Orthod.

